# *ERCC6L2*‐associated inherited bone marrow failure syndrome

**DOI:** 10.1002/mgg3.388

**Published:** 2018-04-06

**Authors:** Iren Shabanova, Elisa Cohen, Michaela Cada, Ajoy Vincent, Ronald D. Cohn, Yigal Dror

**Affiliations:** ^1^ Genetics and Genome Biology Program The Hospital for Sick Children Toronto ON Canada; ^2^ Marrow Failure and Myelodysplasia Program Division of Hematology/Oncology Department of Pediatrics The Hospital for Sick Children Toronto ON Canada; ^3^ Department of Ophthalmology and Vision Sciences The Hospital for Sick Children Toronto ON Canada; ^4^ Division of Clinical and Metabolic Genetics Department of Paediatrics The Hospital for Sick Children Toronto ON Canada; ^5^ Department of Molecular Genetics The Hospital for Sick Children Toronto ON Canada; ^6^ Institute of Medical Sciences University of Toronto Toronto ON Canada

**Keywords:** bone marrow failure, developmental delay, *ERCC6L2*, microcephaly, thrombocytopenia

## Abstract

**Background:**

*ERCC6L2‐*associated disorder has recently been described and only five patients were reported so far. The described phenotype included bone marrow, cerebral, and craniofacial abnormalities. The aim of this study was to further define the genetic and phenotypic spectrum of the disorder by summarizing the five published cases and an additional case that we identified through whole‐exome sequencing performed at the University of Toronto.

**Methods:**

Clinical data was extracted from the Canadian Inherited Marrow Failure Registry. Whole exome sequencing was performed to identify causative mutations**.**

**Results:**

All six cases had homozygous truncating mutations either at or upstream of the helicase domain of *ERCC6L2*. All patients displayed bone marrow failure, learning or developmental delay and microcephaly. Our patient was unique in displaying features of cerebellar disease, including ataxia and dysmetria as well as an interval deterioration of the corpus callosum and generalized volume loss on MRI. Another unique feature of our patient was retinal dystrophy with macular involvement. Along with one other patient, our patient displayed craniofacial abnormalities by presenting with low‐set prominent ears, a pointed prominent chin, and deep‐set eyes. Leukemia is common among patients with inherited bone marrow failure, but thus far, none of the patients have developed this complication.

**Conclusions:**

*ERCC6L2‐*associated disorder is a multisystem disorder. The phenotype spectrum includes bone marrow failure, cerebral, and craniofacial abnormalities, as well as cerebellar and retinal abnormalities.

## 1 INTRODUCTION

*ERCC6L2*‐associated disorder has recently been described. The prevalence of the disorder is unknown; however, the disorder is probably very rare, and thus far, only five cases have been reported (Tummala et al., 2014; Zhang et al., 2016; Jarviaho et al., 2017). The spectrum of clinical manifestations and mutations is yet to be defined, but all the reported patients had bone marrow failure, and three of the given patients had both microcephaly and developmental delay. Herein, we describe in detail a female patient with a novel pathogenic homozygous mutation in *ERCC6L2* (OMIM 615667) with several new clinical features that have not been previously reported. In addition, we summarize the findings of all six identified cases and update the spectrum of phenotypes and genotypes of this rare syndrome.

## 2 CASE DESCRIPTION

The patient in this report was enrolled on the Canadian Inherited Marrow Failure Registry and informed written consent was obtained. The study was approved by the Hospital for Sick Children's Research Ethics Board. The patient was a female, who presented at the age of two months with poor weight gain, requiring G‐tube feeding and a height below the 3rd percentile. After 6 months of age, her head circumferences were noticed to be below the 3rd percentile. Previous evaluations of the child did not report microcephaly, but specific measurements were not available. Since about 6 months of age, the child was also noticed to have developmental delay. Other features noted on physical examination included low set prominent ears, a pointed prominent chin, deep‐set eyes and one café au lait spot on the abdomen, which was about 1.5 × 1.5 cm. Neurological examination showed hypertonia, brisk deep tendon reflexes, bilateral clonus, down‐going plantar reflexes, strabismus, and features of cerebellar disease that included dysmetria, ataxia and nystagmus. At 3½ years old, triple‐evoked potentials and somatosensory‐evoked potentials of the median nerve showed evidence of central conduction delay.

Brain MRI at the age of 6 years showed diffuse hazy T2 signal of hyperintensity throughout the supratentorial white matter. Repeat MRI at 10 years old displayed an interval increase in supratentorial FLAIR hyperintensity, involving the posterior limbs of the internal capsule, cerebral peduncle, external capsule, peritrigonal white matter, and optic radiation. There appeared to be reduced white volume with mild thinning of the corpus callosum and overall generalized supra and infratentorial volume loss.

On eye examination, the macula showed patches of atrophy, whereas the rest of the retina showed generalized pigment epithelial changes; electroretinography showed generalized rod‐cone dystrophy with additional selective involvement of the retinal ON‐pathway. Visual‐evoked potentials showed evidence of central conduction delay.

The patient had persistent mild thrombocytopenia and intermittent mild neutropenia. At 8½ years of age, her complete blood counts showed platelets 58 × 10^9^/L, WBC 3.0 × 10^9^/L, neutrophils 1.1 × 10^9^/L, Hb 133 g/L, MCV 88.9 fl, absolute reticulocyte count of 64.9 × 10^9^/L, and hemoglobin F of 3.7%. The patient's bone marrow testing showed severe hypocellularity of <10%–20% with reduced trilineage hematopoiesis.

The patient underwent multiple investigations to reveal the cause for the above manifestations which all showed normal results, including testing for: karyotype, fragile X by FMR1 (CGG)n repeat, a metabolic screen, congenital disorders of glycosylation, mitochondrial disorders, alpha fetoprotein, chromosome breakage studies (for Fanconi Anemia), targeted sequencing of *FXN* (Friedreich Ataxia), *SBDS* (Shwachman‐Diamond syndrome), *MECP2* (Rett syndrome), *ERCC6* and *ERCC8* (Cockayne syndrome), and copy number variant analysis by oligonucleotide microarray. A microcephaly panel, which sequenced 41 known genes in autosomal recessive microcephaly, was also tested and found to be negative. Testing for Ataxia Telangiectasia, Nijmegen Breakage syndrome, and Bloom syndrome by chromosome analysis were all negative. Telomere length (for dyskeratosis congenita) of total lymphocytes and memory T cells and B cells was at the 1st percentile; length of telomere in granulocytes and natural killer cells was below the 1st percentile. These findings were not typical of dyskeratosis congenita.

At the age of 9 years, the patient underwent whole exome sequencing and was discovered to have a homozygous stop mutation in *ERCC6L2* (NCBI RefSeq NG_034107.1)*,* c.1687C>T (p.Arg563*). No other causal mutations were identified.

## 3 DISCUSSION

**Figure 1:**
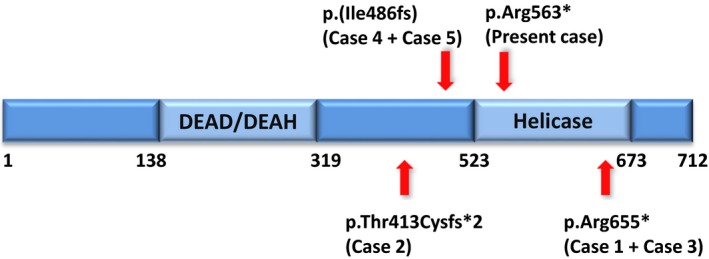
Structure of the ERCC6L2 protein. The locations of patients’ mutations (Cases 1‐6) are indicated

Together with the present case, there are six published cases of patients with an *ERCC6L2‐*related disorder. The features of these six patients are summarized in Table 1. The addition of our case further supports the link between *ERCC6L2* and this recently characterized syndrome. All six cases were caused by truncating mutations either at or upstream of the helicase domain leading to premature termination of translation (Figure 1). Since *ERCC6L2* belongs to the Snf2 family of helicase‐related proteins, it is possible that deletion of the helicase domain is sufficient to cause the disease due to the importance of the Snf2 family of helicase‐related proteins in regulation of nuclear processes (Tummala et al., 2014; Flaus, Martin, Barton, & Owen‐Hughes, 2006). Alternatively, a complete loss of the protein due to either nonsense‐mediated mRNA decay or protein degradation by the ubiquitin‐proteasome system is the cause of the phenotype. Protein degradation by the ubiquitin‐proteasome system plays an important role in several fundamental cellular processes, including regulation of the cell cycle, modulation of cell surface receptors and ion channels, as well as antigen presentation and has been linked to several hereditary human diseases (Sarikas et al., 2005).

**Table 1:** Clinical and genetic characteristics of the six known cases with ERCC6L2‐associated disorder

Features	Case 1 (Family 1) (Tummala et al., 2014)^a^	Case 2 (Family 2) (Tummala et al., 2014)^b^	Case 3 (Family 3) (Zhang et al., 2016)^c^	Case 4 (Family 4) (Jarviaho et al., 2017)^d^	Case 5 (Family 5) (Jarviaho et al., 2017)^e^	Case 6 (Family 6) (Present study)^f^
Gender	Male	Female	Male	Male	Female	Female
Age at presentation (years)	12	19	13	8	8	7
Ethnic origin	French	Pakistani	UnK	Finnish	Finnish	Pakistani
First‐cousin parents	Yes	Yes	Yes	No	No	Yes
Platelet counts (×10^9^/l)	9	33	Initially 4; then improved to moderate level of thrombocytopenia	5	23	58
Hemoglobin (g/l)	99	93	Initial: 90 Resolution: 120	81	106	133
Reticulocytes (×10^9^/l)	UnK	UnK	75 (At time of improved counts)	UnK	UnK	64.9
MCV (fl)	UnK	UnK	104 (initially) 104 (at the time of improved counts)	UnK	UnK	88.9
WBC (x10^9^/l)	2.4	5.3	1.47 (at the time of improved counts)	2.3	2.0	3.0
Neutrophils (×10^9^/l)	UnK	UnK	0.7 (initially) Then improved to moderate neutropenia	UnK	UnK	1.1
HbF	UnK	UnK	7%^g^	UnK	UnK	3.7%^g^
Bone marrow cellularity	Hypocellular	Hypocellular	Hypocellular	Hypocellular	Reduced cellularity	Hypocellular
Course of bone marrow failure	UnK	UnK	Spontaneous recovery	UnK	UnK	Stable over 3 years
Developmental delay and/or learning difficulties	Yes	Yes	Yes	No	No	Yes
Microcephaly	Yes	Yes	Yes	No	No	Yes
Signs of upper motor neuron palsy	UnK	UnK	UnK	UnK	UnK	Hypertonia
Cerebellar features	UnK	UnK	UnK	UnK	UnK	Ataxia, dysmetria, nystagmus
Hypotonia	UnK	During infancy	UnK	UnK	UnK	UnK
Eyes (internal compartments)	UnK	UnK	UnK	UnK	UnK	Rod and cone dystrophy,
Craniofacial abnormalities	Abnormal facies and ear abnormalities	UnK	UnK	UnK	UnK	Low set prominent ears, pointed prominent chin, deep‐set eyes
Kidneys	UnK	UnK	Bilateral pyeloureteral junction abnormalities	UnK	UnK	UnK
Height and weight	UnK	UnK	<1 SD for weight; <0.5 SD for height	−2.7 SD	UnK	UnK
Vascular	UnK	UnK	Vascular abnormalities in right frontal lobe	UnK	UnK	UnK
Other clinical features	None	None	None	Vitamin B12 deficiency and pubertal delay	None	None
Chromosomal breakage with and without MMC and DEB	Normal	Normal	UnK	UnK	UnK	Normal
Telomere length	Normal	Short	UnK	Normal	Normal	Short
*ERCC6L2* mutation	c.1963 C>T p.Arg655*	c.1236_1239delAACA p.Thr413Cysfs*2	c.1963 C>T p.Arg655*	c.1457del p.(Ile486 fs)	c.1457del p.(Ile486 fs)	c.1687C>T p.Arg563*^,^ h

a Peripheral‐blood analysis at presentation showed hemoglobin at 99 g/l, white cell count at 2.4 × 10^9^/L, platelets at 9 × 10^9^/L, and very hypocellular bone marrow.

b Peripheral‐blood analysis at presentation showed hemoglobin at 93 g/l, white cell count at 5.3 × 10^9^/L, platelets at 33 × 10^9^/L, and hypocellular bone marrow with features of dysplasia.

c *Initial CBC's revealed*: thrombocytopenia (4 × 10^9^/L), mild anemia (Hb 90 g/L), macrocytosis (MCV 104 fl), moderate neutropenia (neutrophils 0.7 × 10^9^/L*); Then spontaneously resolved to*: Hb 120 g/L, MCV 104 fl, reticulocytes of 75 × 10^9^/L, moderate neutropenia, thrombopenia, and low lymphocyte count (1.47 × 10^9^/L).

d Peripheral‐blood analysis at 15 years of age showed hemoglobin 81 g/L, white cell count 2.3 × 10^9^/L, platelets 5 × 10^9^/L, and hypocellular bone marrow.

e Peripheral‐blood analysis at 11 years of age showed hemoglobin 106 g/L, white cell count 2.0 × 10^9^/L, platelets 23 × 10^9^/L, and reduced cellularity in bone marrow.

f Peripheral‐blood analysis at presentation showed hemoglobin at 130 g/l, white cell count at 4. 7 × 10^9^/L, platelets at 82 × 10^9^/L, and hypocellular bone marrow.

g The values are high for age.

h The patient also has biallelic mutations in *C2Orf71* that is associated with retinitis pigmentosa. The variants are c.3739G>A (pGly1247Ser) and c.1882G>A (p.Ala628Thr). Each of the variants was inherited from a different parent.

TEP, triple evoked potentials; UnK, unknown.

All six cases manifested hematopoietic features. Thrombocytopenia was the most prominent phenotypic feature and was seen in all cases; it was moderate in two cases and severe in four cases. Anemia was seen in five cases and ranged from mild to moderate. In one case, reticulocyte counts were available when the patient was anemic and were low for the degree of anemia. Hemoglobin F was tested in two cases and was elevated at 7% and 3.7%. Neutrophils were reported in two cases and were moderately low in both of them. MCV was reported in two cases and was elevated in one patient. Bone marrow was hypocellular in all cases. Myelodysplastic syndrome and leukemia were not reported among the six patients; however, due to the small series and the predicted functions of *ERCC6L2*, it is reasonable to manage these patients with a leukemia surveillance program until a leukemic risk is confidently excluded. Importantly, most syndromes with inherited bone marrow failure are leukemia prone (Dror, 2017). Therefore, establishing a diagnosis in such cases immediately changes the management of these patients and a leukemia surveillance program is initiated. Further, a significant proportion of patients with inherited bone marrow failure syndromes may not manifest prominent bone marrow failure before developing leukemia. Hence, inclusion of *ERCC6L2* in the list of genes to be tested when the features described in the present manuscript are encountered may be the only tool that allows for early institution of a leukemia‐surveillance program.

The neuronal presentation was noteworthy with four patients showing learning difficulties and developmental delay. Microcephaly was also present in four of the cases. In our case, nerve conduction velocity was measured which showed central delay. Our patient was unique in displaying features of cerebellar disease, including ataxia and dysmetria. Importantly, in our patient, we have noticed an interval deterioration of the corpus callosum and generalized volume loss on MRI. This suggests the possibility of a neurodegenerative process. Nevertheless, the patient has not shown clinical signs of deterioration.

Two of the patients presented with craniofacial abnormalities, while information on the other four patients was not provided. Case 1 displayed “abnormal facies” and ear abnormalities, while our patient had low‐set prominent ears, a pointed prominent chin, and deep‐set eyes.

The patient in the present report also had early onset retinal dystrophy with macular involvement. The patient had compound heterozygous variants in *C2orf71,* a gene associated with retinitis pigmentosa, of (c.3739G>A (pGly1247Ser) and c.1882G>A (p.Ala628Thr)). The reported minor allele frequencies of the variants in the GnomAD database were 3% (p.Gly1247Ser) and 1.1% (p.Ala628Thr) among South Asians, and both variants were also reported in a homozygous state in the general population. Further, bioinformatics analysis showed inconsistent results with regard to potential damaging effect. Thus, these variants were excluded as a cause of the retinal phenotype. Interestingly, retinitis pigmentosa was associated with several other hereditary bone marrow failure disorders, including *DNAJC21*‐related Shwachman‐Diamond syndrome (Tummala et al., 2014; Dhanraj et al., 2017) and Revesz syndrome (Savage et al., 2008). Therefore, it is possible that retinitis pigmentosa is part of the phenotypic spectrum of the *ERCC6L2*‐related disorder or the disorder is caused by a variant(s) that was not detected by whole exome sequencing (Dhanraj et al., 2017). Reports of additional cases of *ERCC6L2*‐related disorder are needed to determine a causal relationship.

The *ERCC6L2* gene locus is on chromosome 9 (9q22.32) and spans 14 exons. It has two distinct domains: an N‐terminal DEAH ATP‐helicase domain, and a catalytic helicase C‐terminal domain. *ERCC6L2* belongs to the Snf2 family of helicase‐like proteins, which are involved in chromatin unwinding, transcription regulation, and DNA recombination, translocation, and repair (Flaus et al., 2006). Most importantly, it was proposed to play a role in the DNA‐damage response. Since a DNA‐damage response is required during cell proliferation and tissue maintenance, *ERCC6L2*‐deficiency would result in a slow accumulation of DNA damage. In response to genotoxic stress, *ERCC6L2* genes translocate from the cytosol to both the mitochondria and nucleus (Tummala et al., 2014). Knockdown of *ERCC6L2* genes in lung cancer cells showed an increase in DNA damage and intracellular ROS. Zhang and colleagues have identified a new protein, HEBO, which is formed from alternative splicing of *ERCC6L2*. HEBO is a DNA repair factor localized mainly in the nucleus that was recruited to sites of DNA lesions in an NBS1‐dependent manner (Zhang et al., 2016). The authors identified a patient with an *ERCC6L2* homozygous nonsense mutations (Case 3 in Table 1) that results in truncation of about half of the HEBO.

In summary, the data collected from cases of patients with biallelic mutations in *ERRC6L2* results in a unique syndrome that includes bone marrow failure, cerebral and cerebellar abnormalities, as well as craniofacial abnormalities. The addition of new cases allows for opportunities to further study this bone marrow failure syndrome and continue defining its pathogenesis and phenotypic spectrum.

## CONFLICT OF INTERESTS

None declared.
